# Frequency of Hepatobiliary Manifestations and Concomitant Liver Disease in Inflammatory Bowel Disease Patients

**DOI:** 10.1155/2019/7604939

**Published:** 2019-01-31

**Authors:** Juliana Silva, Beatriz S. Brito, Isaac Neri de N. Silva, Viviane G. Nóbrega, Maria Carolina S. M. da Silva, Hemerson Dyego de N. Gomes, Flora Maria Fortes, Andrea M. Pimentel, Jaciane Mota, Neogélia Almeida, Valdiana C. Surlo, André Lyra, Raquel Rocha, Genoile O. Santana

**Affiliations:** ^1^Department of Life Sciences, Universidade do Estado da Bahia, Rua Silveira Martins 2555, Cabula 41150-000, Salvador, Bahia, Brazil; ^2^Hospital Geral Roberto Santos (HGRS), Rua Direta do Saboeiro s/n, Cabula - CEP: 41180-780, Salvador, Bahia, Brazil; ^3^Gastroenterology Unit, University Hospital Professor Edgard Santos, Universidade Federal da Bahia, Rua Augusto Viana sn/28 andar, Canela 40110-060, Salvador, Bahia, Brazil; ^4^Departamento Ciência da Nutrição, Escola de Nutrição, Universidade Federal da Bahia, Avenida Araújo Pinho, 32, Canela 40110-150, Salvador, Bahia, Brazil

## Abstract

**Background:**

In inflammatory bowel disease (IBD) patients there are reports of the occurrence of hepatobiliary manifestations, so the aim of this study was to evaluate the hepatobiliary manifestations in patients with Crohn's disease (CD) and ulcerative colitis (UC) from an IBD reference center.

**Methods:**

Cross-sectional study in an IBD reference center, with interviews and review of medical charts, between July 2015 and August 2016. A questionnaire addressing epidemiological and clinical characteristics was used.

**Results:**

We interviewed 306 patients, and the majority had UC (53.9%) and were female (61.8%). Hepatobiliary manifestations were observed in 60 (19.6%) patients with IBD. In the greater part of the patients (56.7%) hepatobiliary disorders were detected after the diagnosis of IBD. In UC (18.2%) patients, the hepatobiliary disorders identified were 11 (6.7%) non-alcoholic fatty liver disease, 9 (5.5%) cholelithiasis, 6 (3.6%) primary sclerosing cholangitis (PSC), 3 (1.8%) hepatotoxicity associated with azathioprine, 1 (0.6%) hepatitis B, and 1 (0.6%) hepatic fibrosis. In CD (21.3%) patients, 11 (7.8%) had cholelithiasis, 11 (7.8%) non-alcoholic fatty liver disease, 4 (2.8%) PSC, 3 (2.1%) hepatotoxicity, 1 (0.7%) hepatitis B, (0.7%) hepatitis C, 1 (0.7%) alcoholic liver disease, and 1 (0.7%) autoimmune hepatitis (AIH). There was one case of PSC/AIH overlap syndrome.

**Conclusion:**

The frequency of hepatobiliary disorders was similar in both forms of IBD in patients evaluated. The most common nonspecific hepatobiliary manifestations in IBD patients were non-alcoholic liver disease and cholelithiasis. The most common specific hepatobiliary disorder was PSC in patients with extensive UC or ileocolonic CD involvement; this was seen more frequently in male patients.

## 1. Introduction

Ulcerative colitis (UC) and Crohn's disease (CD) are inflammatory bowel diseases (IBD) that have different clinical presentations and are responsible for chronic idiopathic inflammation of the intestine. Several extraintestinal manifestations may be associated with IBD, and the disease may have a profound impact on patients' quality of life [1, 2].

Hepatobiliary extrahepatic manifestations are sometimes underdiagnosed in IBD patients, and their presence may impair the patients' prognosis [3]. A few studies from Brazil have evaluated the presence of liver extrahepatic manifestations associated with IBD. Approximately 30% of IBD subjects present with increased liver enzymes, and this may represent a diagnostic challenge [4]. Of note, IBD patients may present with liver diseases that are not necessarily associated with the intestinal illness.

Non-alcoholic fatty liver disease (NAFLD) is the liver disease most commonly found in patients with IBD that is not linked to intestinal activity [3]. On the other hand, primary sclerosing cholangitis (PSC) is the most common specific liver disease associated with IBD, especially with ulcerative colitis (UC), and it occasionally may overlap with autoimmune hepatitis. The hepatobiliary disorders less frequently associated with IBD are autoimmune hepatitis/PSC overlap syndrome: IgG4-associated cholangiopathy, primary biliary cholangitis, hepatic amyloidosis, granulomatous hepatitis, cholelithiasis, portal vein thrombosis, and hepatic abscess. The spectrum of these manifestations varies according to the type of IBD. Granulomas, hepatic abscess, amyloidosis, and gallstones are traditionally observed in CD, while PSC and autoimmune hepatitis are usually described in UC patients [5, 6].

IBD therapy may also be associated with hepatic toxicity [6]. Knowledge of the prevalence of hepatitis B and hepatitis C in patients with CD is of fundamental importance, since viral replication may occur during immunosuppressive therapy [7].

Patients with some hepatobiliary manifestations may progress to severe hepatic dysfunction and the need for liver transplantation. PSC patients are more likely to develop cholangiocarcinoma and colon cancer. Therefore, early recognition and better characterization of these manifestations are of fundamental importance to develop the appropriate clinical management and public health policies. The present study aimed to describe the hepatobiliary manifestations of patients with IBD.

## 2. Methods

This was an observational cross-sectional study performed at the Inflammatory Bowel Disease Outpatient Clinic of the Hospital Geral Roberto Santos (HGRS), which is a reference center for IBD.

Data collection was done with the use of a questionnaire addressing epidemiological and clinical characteristics and, mainly, hepatobiliary involvement; data were also pulled from patient medical records. Our cohort, which we followed during the period from June 2015 to August 2016, included patients with IBD over the age of 18 years. Subjects who were not able to respond to the questionnaire or who had unclassified colitis were excluded.

Data were collected by reviewing the records of patients who signed the informed consent term after agreeing to participate in the investigation. The study was approved by the Research Ethics Committee.

The analyzed variables included provenance (rural or urban zone); genre; age; ethnicity/color; alcoholism; age (A): less than or equal to 16 years old in diagnosis (A1), 17 to 40 years old (A2), and greater than 40 years old (A3); location (L): ileal (L1), colonic (L2), ileocolonic (L3), and upper gastrointestinal tract (L4); and behavior (B): inflammatory (B1), stenosing (B2), and penetrating/fistulizing (B3), in addition to the presence or absence of perianal disease (P) [8].

The frequency of hepatobiliary manifestations included cholelithiasis, chronic viral hepatitis B and C, liver enzyme alterations with no defined etiology, primary sclerosing cholangitis (PSC), non-alcoholic fatty liver disease (NAFLD), pericholangitis, cholelithiasis, cholangiocarcinoma, autoimmune hepatitis (AIH), alcoholic liver disease, hepatic amyloidosis, hepatic abscess, primary biliary cirrhosis, granulomatous hepatitis, and portal vein thrombosis.

Laboratory variables analyzed included AST, ALT, gamma-GT, alkaline phosphatase, bilirubin, prothrombin time, albumin, and the hepatitis viral markers HBsAg, anti-HBC IgM, anti-HBC IgG, and anti-HCV.

Imaging variables analyzed included abdominal ultrasound, magnetic resonance imaging of the abdomen, and endoscopic retrograde cholangiopancreatography. Also assessed were IBD pharmacological treatment, hepatic biopsy, and autoantibodies when present (ASCA and p-ANCA).

Mean, standard deviation of the numerical variables, and categorical variables frequencies were analyzed using the statistical package SPSS version 21.0. Categorical variables were analyzed using the chi-square test, and continuous variables were analyzed using Student's t-test. Differences were considered statistically significant when the probability of type 1 error was <0.05.

## 3. Results

In this study, 306 patients with an IBD diagnosis were interviewed (165 UC, 141 CD).  Table 1 shows the demographic characteristics of the patients with IBD with and without hepatobiliary manifestations. Among the 60 patients who had hepatobiliary manifestations, 30 (50.0%) had UC. The same patient might have had more than one manifestation.

The frequency of hepatobiliary manifestations in IBD patients is described in Table 2. Hepatic amyloidosis, hepatic abscess, primary biliary cholangitis, cholangiocarcinoma, pericholangitis, granulomatous hepatitis, and portal vein thrombosis were not detected.

Hepatobiliary symptoms detected at the time of the interview included jaundice in 1.3% (n=4) of patients (notably, two of them had no diagnosis of hepatobiliary disease); choluria in 1.0% (n=3) of patients (likewise, two patients had no diagnosis of hepatobiliary disease); pruritus in 3.5% (n=12) of patients (six of whom had no confirmed hepatobiliary disease); and pain in the right upper quadrant in 8.5% (n=26) of patients (17 of whom were without a diagnosis of hepatobiliary disease). We did not find signs of hepatic cirrhosis.

Liver blood tests were altered in 20.3% of the patients, including elevations of aminotransferases, canalicular enzymes, or bilirubin. For patients with CD, 128 of 141 had liver profile exams; 22.7% (n=29) presented with alterations in aminotransferases, canalicular enzymes, or bilirubin; of these, 27.6% (n= 8) had a diagnosis of some concurrent hepatobiliary manifestations, and 72.4% (n=21) had no hepatobiliary disease diagnosis defined until the time of evaluation.

PSC prevalence among men in both the UC and CD groups was of 6.2% among men with UC and 2% in women with UC and 5.8% among men with CD and 2% in women with CD.

The temporal relationship between the presentation of hepatobiliary manifestations and the diagnosis of IBD is described in Table 3.

The Montreal classification (2005) for UC and CD is described in Tables 4, 5 and 6.

Hepatitis B serology analysis of 182 patients including those already diagnosed with hepatitis B showed a positive HBsAg in 2 subjects (1.1%); 2.7% were isolated IgG HBcAb positive. HBs antibody was performed in 79 patients who also were IgG HBcAb positive, and 68.0% (n=46) were unprotected for hepatitis B. Anti-HCV was obtained in 181 patients, being positive in 2.2% (n=4) of the cases.

## 4. Discussion

It is important to evaluate the frequency of hepatobiliary manifestations in patients with IBD due to the risk of progression to chronic liver disease and possible association with neoplastic diseases. In our study, we obtained a frequency of 19.6% of hepatobiliary manifestations in patients with IBD, considering viral hepatitis cases and drug hepatotoxicity, in addition to specific or associated IBD diseases. The presence of hepatobiliary manifestations was similar between patients with CD and UC, and their epidemiological profile did not differ from that of other patients with IBD, the majority of whom were female, from the urban area and of African descent. The mean age was higher for patients with hepatobiliary manifestations compared to those without hepatobiliary manifestations.

Our results corroborate with the literature regarding the moment of diagnosis of hepatobiliary alterations in patients with IBD. A previous study showed an overall proportion of 11.2% of cases diagnosed prior to IBD, 19.2% concomitant and 69.6% thereafter [9].

In the study by Lakatos et al. [10], the frequency of hepatobiliary manifestations was 12.4% in UC patients and 22.4% in those with CD; this lower frequency of hepatobiliary manifestations observed in the UC group might be explained by the fact that the study by Lakatos et al. [10] evaluated only PSC, small duct PSC, and NAFLD.

PSC is considered to be of great importance due to its severity and the risk for the patient of developing cholangiocarcinoma and colorectal cancer. The study of Vries et al. [11] reported a prevalence of approximately 2.4% to 7.5% in patients with UC and 3.4% in patients with CD, similar data to ours. According to Saich et al. [5] the disease is rare in the general population but is strongly associated with IBD, affecting up to 5% of patients with UC and 3.6% with CD. Other studies described the prevalence of PSC association with UC of 8%, compared to 1% to 3% in CD [12].

PSC is a chronic inflammatory disease that is highly associated with IBD characterized by intra- and extrahepatic bile ducts stenosis, fibrosis, and obstruction. IBD-associated PSC has an increased frequency among patients with extensive colitis, and its course may range from mild symptoms to more severe disease. The presence of IBD associated PSC increases the chance of the development of colorectal cancer by up to 10-fold compared to control groups with UC [13]. Claessen et al. [14] reported that patients with IBD-associated PSC are at high risk for colorectal cancer and that this risk is approximately three times higher than that of cholangiocarcinoma. According to the systematic review by de Vries et al. [11] the prevalence of IBD among PSC patients varies from 46.5% to 98.7% and is more frequent in UC subjects. On the other hand, CD is found in approximately 16% of patients with IBD; the association with undetermined colitis is the least common, rarely accounting for more than 5% of cases.

Evidence suggests the prevalence of PSC in IBD varies according to the extent of disease; the prevalence of PSC is approximately 5.5% in patients with extensive colitis but only 1% in subjects with left colitis [12]. The distribution of patients with UC and PSC shows that, for most, the entire colon is involved: extensive colitis (64.7%), left colitis (18.8%), and proctitis (1% to 5.5%). In PSC patients with CD, colon involvement is reported most often (36.8%-82.1%), followed by involvement of the ileum and colon (21.8%-57.9%). Isolated ileal involvement is rare (2%-5%) [11].

In a study that analyzed 262 patients with CD, 15% had abnormal liver function tests; they underwent ERCP and liver biopsy, and 3.4% (n=9) presented with PSC [15]. CD-associated PSC usually presents with extensive colonic or ileocolonic diseases, as well as in pediatric populations [5]. In Italy, Zippi et al. [9] analyzed 216 patients with CD and 595 with UC and found only 0.9% (n=2) of cases of PSC in CD patients and 0.7% in UC subjects. These studies showed a lower frequency of PSC than did ours. We do not know if this finding can be attributed to the sample size or to the characteristics of the populations studied.

A large population-based study about IBD extraintestinal manifestations conducted in Canada by Bernstein et al. [16] included 4454 patients and found a 2% PSC prevalence in UC patients and a 0.4% prevalence in those with CD. Men were more commonly affected in the UC group (3% vs 1% of women) and in the CD group (0.4% vs 0.3% of women). Wang et al. [12] showed result similar to ours, with a mean age at the time of PSC diagnosis of 40 years and a slight male predominance [17].

Studies have described AIH-associated UC or pediatric cases. UC may be present in 16% of AIH cases [18]. The frequency of PSC and AIH coexistence ranges from 7.6% to 53.8% of patients with PSC [5]. Studies have suggested that patients with AIH associated with IBD are more likely to have disease recurrence and progression to cirrhosis [6]. In our study, there was one case (0.3%) of autoimmune hepatitis overlapping with PSC in a CD patient.

The prevalence of hepatic steatosis varies worldwide and has been reported to be 18% in our region, according to the study of Matteoni et al. [19], who used ultrasonography evaluation in all patients. In our study, the lower prevalence of NAFLD might be attributed to the constitutional characteristics of the studied population, although it is interesting to speculate that IBD could somehow interfere with the development of NAFLD. The coexistence of NAFLD with IBD has gained epidemiological importance. Its prevalence in other studies varies from 6.2%, similar to our study, to 40% [6]. A recent study showed a higher prevalence rate of NAFLD in patients with IBD compared to the incidence of the general population of 9.1/100 in patients with IBD and of 0.029 to 3.1 in the general population. The factors that led to this increase in IBD are uncertain. Some studies point to metabolic syndrome as the main factor in these patients; however it is still a controversial issue [6].

According to a systematic review [6], the prevalence of cholelithiasis ranges from 11% to 34% in CD, and hepatobiliary manifestations are more common in CD patients. A study from Stockholm, which compared the occurrence of gallstones in the general population and in patients with CD, showed a relative risk of 1.8 for patients with CD [3]. This difference compared to our study might be explained by the fact that the cited studies are cohort- and hospital-based, unlike ours, which is transverse and outpatient-based. In the study by Parente et al. [20], among patients with IBD, a cholelithiasis frequency of 9.9% was found in the CD group, while in the UC group, it was 7%, in agreement with our results.

Risk factors, including use of total parenteral nutrition (NPT) and frequency and duration of hospitalizations, while important, were not analyzed. Most drugs used in IBD are potentially hepatotoxic, although the incidence of serious complications is low [6]. A moderate increase in aminotransferases ranges from 19% to 30% [6]. Hepatic toxicity varies according to the group of studied drugs, and, in theory, all medications used for IBD can lead to liver damage, including amino salicylates, steroids, methotrexate, thiopurines (azathioprine and its main active metabolite 6-mercaptopurine), and anti-TNF and anti-integrin immunobiological agents [6]. Of note, in one of our cases, hepatic injury was caused by methotrexate, an immunosuppressant used to maintain clinical remission of CD. In the other cases, it was not possible to precisely identify the drug used. Methotrexate injury may range from macrovesicular steatosis to liver fibrosis.

We found a frequency of alcoholic hepatitis of 0.7%, emphasizing the impact of certain life habits on the frequency of hepatobiliary manifestations among a few IBD patients. Other studies did not report this variable but however stress it as an important risk factor for cirrhosis [4].

The prevalence of hepatitis B and hepatitis C in IBD patients is similar to that found in the general population [4, 7]. In our study, 38% of the subjects were protected for hepatitis B, 2.3% had prior virus contact, and 0.7% had chronic hepatitis B infection. Other authors have found the rate of positive anti-HBc to be 8.1% and the rate of HBsAg to be 1% among IBD patients [7, 21]. Our results disagree with those of a review of six studies in which the anti-HCV positive rate was 3.3% amid patients with IBD [7]. We did not find cases of viral hepatitis reactivation with immunobiological therapy. This could be due to the systematic screening used before immunosuppressants and anti-TNF utilization in the study population. Our results agree with the general population prevalence of HBsAg and HCV in the Brazilian population.

Finally, the limitations of this study are mostly related to the fact that this is a cross-sectional study without a prospective evaluation, which would be ideal for better case identification. However, the data presented highlight the importance of a systematic and prospective search for hepatobiliary manifestations among IBD patients, given that early diagnosis may contribute to better management of possible complications associated with hepatobiliary diseases.

In conclusion, we found that the presence of hepatobiliary manifestations was frequent in both patients with CD and UC. Most of the epidemiological profile of these patients did not appear to differ from other subjects with IBD, most of whom were female from urban areas. The mean age was higher for patients with hepatobiliary manifestations compared to the general sample. The diagnosis of hepatobiliary manifestations is most commonly performed after the diagnosis of IBD has been established. According to the Montreal classification, the majority of patients with hepatobiliary manifestations who presented with a diagnosis were older than 17 years (A2 or A3). The inflammatory form predominated, and the disease with colon involvement was more common (L2 or L3) in patients with CD or in UC patients with extensive colitis. The most frequent nonspecific hepatobiliary manifestations in patients with IBD were NAFLD and cholelithiasis. PSC was the most frequent specific hepatobiliary manifestation, affecting more male adults in the fourth decade of life that had colonic involvement.

## Figures and Tables

**Table 1:** Demographic characteristics and frequency of hepatobiliary manifestations in patients with inflammatory bowel disease.

Feature	Patients without hepatobiliary manifestations N=246	Patients with hepatobiliary manifestations N=60	P
*IBD type, n (%)* UC CD	135 (54.9) 111 (45.1)	30 (50.0) 30 (50.0)	0.497
*Sex, n (%)* Female Male	151 (61.4) 95 (38.6)	38 (63.3) 22 (36.7)	0.780
*Mean age (years), mean (SD)*	42.8 (13.7)	48.1 (13.8)	0.009
*Region origin, n (%)* Rural area Urban area	41 (16.7) 203 (82.5)	6 (10.0) 54 (90.0)	0.531
*Ethnicity (self-declared), n (%)* Black White Mixed (“Pardo”) Asia Did not declare	80 (32.5) 30 (12.2) 122 (49.6) 7 (2.8) 7 (2.8)	16 (26.7) 12 (20) 28 (46.7) 2 (3.3) 2 (3.3)	0.730

IBD: inflammatory bowel disease; UC: ulcerative colitis; CD: Crohn's disease; SD: standard deviation.

**Table 2:** Frequency of hepatobiliary manifestations and concomitant liver disease in UC and CD patients.

Hepatobiliary manifestations	Ulcerative colitis N=165	Crohn's disease N=141	Total N =306
Steatosis/steatohepatitis, n (%)	11 (6.7)	11 (7.8)	22 (7.2)
Cholelithiasis, n (%)	9 (5.5)	11 (7.8)	20 (6.5)
PSC, n (%)	6 (3.6)	4 (2.8)	10 (3.3)
Drug hepatotoxicity,*∗* n (%)	3 (1.8)	3 (2.1)	6 (2.0)
Hepatitis B, n (%)	1 (0.6)	1 (0.7)	1 (0.7)
Hepatitis C, n (%)	1 (0.6)	1 (0.7)	2 (0.7)
Hepatic fibrosis, n (%)	0.6(1)	-	1 (0.3)
HAI, n (%)	-	1 (0.7)	1 (0.3)
Alcoholic liver disease, n (%)	-	1 (0.7)	1 (0.3)

PSC: Primary sclerosing cholangitis; HAI: Autoimmune hepatitis.

Note: In some cases, the same patient presented with more than one manifestation.

**Table 3:** Temporal relationship between the diagnosis of hepatobiliary manifestations and the diagnosis of inflammatory bowel disease.

Time of diagnosis of hepatobiliary manifestations in relation to IBD diagnosis	Frequency n (%)
Preceding	14/60 (23.3)
Concomitant	12/60 (20.0)
After	34/60 (56.7)

IBD: inflammatory bowel disease.

**Table 4:** Montreal classification for patients with ulcerative colitis and hepatobiliary manifestations.

Montreal Classification Disease extension	UC without hepatobiliary manifestations N=135	UC with hepatobiliary manifestations N=30
Proctitis, n (%)	22 (16.3)	4 (13.4)
Left colitis, n (%)	57 (42.2)	10 (33.3)
Extensive colitis, n (%)	45 (33.3)	13 (43.3)
No defined extension, n (%)	11 (8.2)	3 (10.0)

UC: ulcerative colitis.

**Table 5:** Montreal classification for patients with CD and hepatobiliary manifestations.

Montreal Classification	CD without hepatobiliary manifestations N=111	CD with hepatobiliary manifestations N=30
*Age at diagnosis, n (%)*		
A1: ≤16 years old	9 (8.1)	2 (6.7)
A2: 17 - 40 years old	72 (64.9)	14 (46.7)
A3: > 40 years old	30 (27.0)	14 (46.7)
*Location, n (%)*		
(L1) Ileal	20 (18.0)	5 (16.7)
(L2) Colonic	44 (39.7)	11 (36.7)
(L3) Ileocolonic	41 (36.9)	10 (33.3)
L1 + superior GI tract (L4)	1 (0.9)	-
L2 + L4	1 (0.9)	-
L3 + L4	1 (0.9)	-
No location defined	3 (2.7)	4 (13.3)
*Behavior, n (%)*		
(B1) Inflammatory	43 (38.8)	10 (33.3)
(B2) Stricturing	15 (13.5)	1 (3.3)
(B3) Penetrating	8 (7.2)	3 (10.0)
B1 + perianal disease (p)	15 (13.5)	9 (30.0)
B2 + p	8 (7.2)	4 (13.3)
B3 + p	18 (16.2)	-
No defined behavior	4 (3.6)	3 (10.0)

CD: Crohn's disease.

**Table 6:** Patients with cholelithiasis and Crohn's disease according to ileal involvement.

Feature	Cholelithiasis	
	Yes N=10*∗*	No N=130
*Ileal involvement (L1 or L3), n (%)* Yes No	7 (9.2) 3 (5.4)	69 (90.8) 53 (94.6)
*Ileum resection, n (%)* Yes No	1 (5.3) 9 (7.6)	18 (94.7) 109 (92.4)

L1: ileal; L3: ileocolonic.

*∗*Note: one of the patients with cholelithiasis had no clear location.

## Data Availability

The data used to support the findings of this study are available from the corresponding author upon request.

## References

[1] Cosnes J., Gowerrousseau C., Seksik P., Cortot A. (2011). Epidemiology and natural history of inflammatory bowel diseases. *Gastroenterology*.

[2] Basso P. J., Fonseca M. T., Bonfá G. (2014). Association among genetic predisposition, gut microbiota, and host immune response in the etiopathogenesis of inflammatory bowel disease. *Brazilian Journal of Medical and Biological Research*.

[3] Gizard E., Ford A. C., Bronowicki J.-P., Peyrin-Biroulet L. (2014). Systematic review: The epidemiology of the hepatobiliary manifestations in patients with inflammatory bowel disease. *Alimentary Pharmacology & Therapeutics*.

[4] Rojas-Feria M., Castro M., Suárez E., Ampuero J., Romero-Gómez M. (2013). Hepatobiliary manifestations in inflammatory bowel disease: The gut, the drugs and the liver. *World Journal of Gastroenterology*.

[5] Saich R., Chapman R. (2008). Primary sclerosing cholangitis, autoimmune hepatitis and overlap syndromes in inflammatory bowel disease. *World Journal of Gastroenterology*.

[6] Restellini S., Chazouillères O., Frossard J.-L. (2017). Hepatic manifestations of inflammatory bowel diseases. *Liver International*.

[7] Gisbert J. P., Chaparro M., Esteve M. (2011). Review article: Prevention and management of hepatitis B and C infection in patients with inflammatory bowel disease. *Alimentary Pharmacology & Therapeutics*.

[8] Satsangi J., Silverberg M. S., Vermeire S., Colombel J.-F. (2006). The montreal classification of inflammatory bowel disease: Controversies, consensus, and implications. *Gut*.

[9] Zippi M., Corrado C., Pica R. (2014). Extraintestinal manifestations in a large series of Italian inflammatory bowel disease patients. *World Journal of Gastroenterology*.

[10] Lakatos L., Pandur T., David G. (2003). Association of extraintestinal manifestations of inflammatory bowel disease in a province of western Hungary with disease phenotype: Results of a 25-year follow-up study. *World Journal of Gastroenterology*.

[11] De Vries A. B., Janse M., Blokzijl H., Weersma R. K. (2015). Distinctive inflammatory bowel disease phenotype in primary sclerosing cholangitis. *World Journal of Gastroenterology*.

[12] Wang R., Leong R. W. (2014). Primary sclerosing cholangitis as an independent risk factor for colorectal cancer in the context of inflammatory bowel disease: A review of the literature. *World Journal of Gastroenterology*.

[13] Nakazawa T., Naitoh I., Hayashi K. (2014). Inflammatory bowel disease of primary sclerosing cholangitis: A distinct entity?. *World Journal of Gastroenterology*.

[14] Claessen M. M. H., Lutgens M. W. M. D., Van Buuren H. R. (2009). More right-sided IBD-associated colorectal cancer in patients with primary sclerosing cholangitis. *Inflammatory Bowel Diseases*.

[15] Rasmussen H. H., Fallingborg J. F., Mortensen P. B. (2009). Hepatobiliary dysfunction and primary sclerosing cholangitis in patients with crohn’s disease. *Scandinavian Journal of Gastroenterology*.

[16] Bernstein C. N., Blanchard J. F., Rawsthorne P., Yu N. (2001). The prevalence of extraintestinal diseases in inflammatory bowel disease: A population-based study. *American Journal of Gastroenterology*.

[17] Bambha K., Kim W. R., Talwalkar J. (2003). Incidence, clinical spectrum, and outcomes of primary sclerosing cholangitis in a United States community. *Gastroenterology*.

[18] Perdigoto R., Carpenter H. A., Czaja A. J. (1992). Frequency and significance of chronic ulcerative colitis in severe corticosteroid-treated autoimmune hepatitis. *Journal of Hepatology*.

[19] Matteoni L., Boente L., Soares D. (2011). Nonalcoholic fatty hepatic disease: Relevance of the diagnosis on abdominal ultrasound. *Gazeta Médica da Bahia*.

[20] Parente J. M. L., Coy C. S. R., Campelo V. (2015). Inflammatory bowel disease in an underdeveloped region of Northeastern Brazil. *World Journal of Gastroenterology*.

[21] Chevaux J.-B., Bigard M.-A., Bensenane M. (2009). Inflammatory bowel disease and hepatitis B and C. *Gastroentérologie Clinique et Biologique*.

